# Switching to nevirapine-based regimens after undetectable viral load is not associated with increased risk of discontinuation due to toxicity

**DOI:** 10.7448/IAS.17.4.19794

**Published:** 2014-11-02

**Authors:** Patricia Patterson, Eugenia Socías, Daniel Pryluka, Pablo Lapadula, Héctor Pérez, Pedro Cahn

**Affiliations:** 1 Clinical Research, Fundación Huésped, Buenos Aires, Argentina; 2 Infectología, Sanatorio Otamendi, Buenos Aires, Argentina; 3 División de Infectología, Hospital Fernández, Buenos Aires, Argentina; 4 Infectología, Centro Médico Huésped, Buenos Aires, Argentina

## Abstract

**Introduction:**

Due to its good tolerability, favourable cardiovascular risk-profile, low-pill burden and cost, nevirapine-based regimens are an attractive simplification strategy for patients with suppressed viral load (VL). However, current guidelines recommend caution if nevirapine (NVP) is prescribed in males and females with CD4 counts above 400 or 250 cells/µL, respectively. The aim of this study is to determine the prevalence and risk factors associated with development of toxicity or treatment discontinuation in patients switching to NVP-based regimens.

**Materials and Methods:**

Retrospective chart review of HIV-infected patients with suppressed VL who switched from a PI-based regimen to a NVP-based regimen in four HIV clinics in Argentina, between 1997 and 2013. Bivariate and multivariate analyses were performed to explore factors associated with treatment discontinuation. High CD4 count was defined as CD4-cell count ≥400 or 250 cells/µL in males and females, respectively.

**Results:**

Of 218 patients included, 165 (75.7%) were male; 21 (9.6%) were co-infected with HCV and/or HBV. Median baseline (BSL) CD4 count: 138 cells/µL (IQR: 64–276). At switch, patients had a median age of 38 years (IQR: 33.4–43.8) and had been suppressed for a median of 1.4 years (IQR: 0.6–2.2); 138 patients (63.3%) had high CD4-cell counts: among females, median CD4 count at switch was 462 (IQR: 330–709) cells/µL; among males, 433 (IQR: 305–595) cells/µL. Thirty-six patients (13.5%) presented NVP-related toxicity (30 skin toxicity, 6 hepatic toxicity), 29 (13.3%) discontinued NVP. Median time to development to toxicity: 32 days (IQR: 15–75). In bivariate analysis, chronic hepatitis was the only variable associated with development of toxicity (OR: 2.90, 95% CI 1.08–7.78). In multivariate analysis, no statistical significant associations were observed between either development of toxicity or treatment discontinuation and gender, chronic hepatitis, age or CD4-cell count at BSL or at switch (all p>0.05).

**Conclusions:**

In our study, switching to a NVP-based regimen in patients with undetectable VL was associated with a low incidence of skin or liver toxicity, and treatment discontinuation. Moreover, these were unrelated to the CD4-cell count. Our findings suggest that, in contrast with ART-naïve patients, switching to NVP-based regimens could be a safe strategy for patients with suppressed viremia regardless of the CD4-cell count.

